# MicroRNA-122: A Novel Hepatocyte-Enriched *in vitro* Marker of Drug-Induced Cellular Toxicity

**DOI:** 10.1093/toxsci/kfu269

**Published:** 2014-12-18

**Authors:** Richard Kia, Lorna Kelly, Rowena L. C. Sison-Young, Fang Zhang, Chris S. Pridgeon, James A. Heslop, Pete Metcalfe, Neil R. Kitteringham, Melissa Baxter, Sean Harrison, Neil A. Hanley, Zoë D. Burke, Mike P. Storm, Melanie J. Welham, David Tosh, Barbara Küppers-Munther, Josefina Edsbagge, Philip J. Starkey Lewis, Frank Bonner, Ernie Harpur, James Sidaway, Joanne Bowes, Stephen W. Fenwick, Hassan Malik, Chris E. P. Goldring, B. Kevin Park

**Affiliations:** ^a^MRC Centre for Drug Safety Science, Department of Molecular and Clinical Pharmacology, Sherrington Buildings, Ashton Street, University of Liverpool, Liverpool L69 3GE, UK ^b^Stem Cells for Safer Medicines, 7th Floor, Southside, 105 Victoria Street, London SW1E 6QT, UK ^c^Faculty of Life Sciences, University of Manchester, Manchester M13 9PL, UK ^d^School of Medicine and Dentistry, University of Central Lancashire, Preston PR1 2HE, UK ^e^Centre for Endocrinology and Diabetes, Institute of Human Development, Faculty of Medical and Human Sciences, Manchester Academic Health Science Centre, University of Manchester, Manchester M13 9PT, UK ^f^Endocrinology Department, Central Manchester University Hospitals NHS Foundation Trust, Oxford Road, Manchester M13 9PT, UK ^g^Centre for Regenerative Medicine, Department of Biology and Biochemistry, University of Bath, Bath BA2 7AY, UK, ^h^Takara Bio Europe AB (former Cellartis), Arvid Wallgrens Backe 20, 413 46 Göteborg, Sweden, ^i^School of Life Sciences, The Systems Biology Research Centre, University of Skövde, Box 408, 541 28 Skövde, Sweden, ^j^MRC Centre for Regenerative Medicine, SCRM Building, The University of Edinburgh, Edinburgh Bioquarter, 5 Little France Drive, Edinburgh EH16 4UU, UK, ^k^Newcastle University, Institute of Cellular Medicine, The Medical School, Framlington Place, Newcastle upon Tyne NE2 4HH, UK ^l^Drug Safety and Metabolism, AstraZeneca R&D, Alderley Park, Cheshire SK10 4TG, UK and ^m^North Western Hepatobiliary Unit, Aintree University Hospital NHS Foundation Trust, Longmoor Lane, Liverpool L9 7AL, UK

**Keywords:** hepatocytes, drug-induced liver injury, microRNA, *in vitro* model, cytotoxicity, cell-specific biomarker, bridging biomarker

## Abstract

Emerging hepatic models for the study of drug-induced toxicity include pluripotent stem cell-derived hepatocyte-like cells (HLCs) and complex hepatocyte-non-parenchymal cellular coculture to mimic the complex multicellular interactions that recapitulate the niche environment in the human liver. However, a specific marker of hepatocyte perturbation, required to discriminate hepatocyte damage from non-specific cellular toxicity contributed by non-hepatocyte cell types or immature differentiated cells is currently lacking, as the cytotoxicity assays routinely used in *in vitro* toxicology research depend on intracellular molecules which are ubiquitously present in all eukaryotic cell types. In this study, we demonstrate that microRNA-122 (miR-122) detection in cell culture media can be used as a hepatocyte-enriched *in vitro* marker of drug-induced toxicity in homogeneous cultures of hepatic cells, and a cell-specific marker of toxicity of hepatic cells in heterogeneous cultures such as HLCs generated from various differentiation protocols and pluripotent stem cell lines, where conventional cytotoxicity assays using generic cellular markers may not be appropriate. We show that the sensitivity of the miR-122 cytotoxicity assay is similar to conventional assays that measure lactate dehydrogenase activity and intracellular adenosine triphosphate when applied in hepatic models with high levels of intracellular miR-122, and can be multiplexed with other assays. MiR-122 as a biomarker also has the potential to bridge results in *in vitro* experiments to *in vivo* animal models and human samples using the same assay, and to link findings from clinical studies in determining the relevance of *in vitro* models being developed for the study of drug-induced liver injury.

Despite the development of various hepatic models for use in screening for adverse effects of new drugs and to aid mechanistic understanding of hepatotoxicity, drug-induced liver injury (DILI) in humans remains a significant cause of patient morbidity and mortality, and confers a major burden to the pharmaceutical industry and the regulatory authorities (Davies *et al.*, 2010; Olsen and Whalen, 2009). This is partly due to the major limitations of currently available hepatic models in recapitulating *in vivo* functional and metabolic capabilities of the human hepatocyte, most notably the expression of drug metabolizing proteins such as cytochrome-P450 (CYP) enzymes, and drug transporters which are important for a mechanistic understanding of drug-induced toxicity (Godoy *et al.*, 2013). The most metabolically active *in vitro* hepatic model is freshly isolated human primary hepatocytes, although a myriad of issues limit their application in the *in vitro* study of drug-induced toxicity and safety screening (Kia *et al.*, 2013). Human primary hepatocytes are not readily available, they are expensive, exhibit large donor variations, and rapidly lose their functional phenotype over time in *in vitro* culture, leading to reduced expression of the majority of CYP enzymes (Godoy *et al.*, 2013; Rodriguez-Antona *et al.*, 2002; Rowe *et al.*, 2010).

A potential new hepatic model is the use of human pluripotent stem cells to generate hepatocytes *in vitro* (Baxter *et al.*, 2010; Bone *et al.*, 2011; Brolen *et al.*, 2010). Directed differentiation of human pluripotent stem cells into hepatocytes, typically called hepatocyte-like cells (HLCs), with a mature functional phenotype, could in theory provide a readily available source of metabolically competent cells for use in drug screening (Greenhough *et al.*, 2010; Yildirimman *et al.,* 2011). However, the differentiation efficiency of HLCs from human pluripotent stem cells can be variable, which is believed to be mainly due to differences of the differentiation protocols being employed and the propensity of the selected pluripotent stem cell line to differentiate toward a hepatic lineage (Baxter *et al.,* 2010; Bock *et al.,* 2011). The differentiation efficiency of HLCs from a starting culture of undifferentiated pluripotent stem cells can range from 9% to 90%, as determined by the percentage of cells in the culture that express the hepatocyte protein marker albumin (Hay *et al.*, 2008; Rashid *et al.,* 2010; Shiraki *et al.*, 2008). Therefore, for the application of HLCs as an *in vitro* model for drug screening and toxicology, this heterogeneity of maturity needs to be accounted for.

Another approach taken to develop a relevant and functional hepatic model includes efforts to better emulate the *in vivo* liver tissue environment that mimics complex multicellular and cell–matrix interactions. Examples include the coculture of primary hepatocytes with non-parenchymal cells such as hepatic sinusoidal endothelial cells and fibroblasts, in either conventional 2-dimensional (2D) platforms or as 3-dimensional (3D) spheroids (Bader *et al.,* 1996; Bhatia *et al.,* 1999). More recently, a complex 3D quasi-liver “bud” was also successfully engineered from a coculture of human pluripotent stem cell-derived HLCs with non-hepatocyte cell lines, and this showed promising functional improvement of the HLCs compared with conventional 2D culture (Takebe *et al.,* 2013).

However, for the application of HLC cultures with heterogeneous maturity and complex hepatic coculture models in the study of drug-induced toxicity, a hepatocyte-specific marker of hepatocyte perturbation is needed to discriminate non-specific cellular toxicity contributed by non-hepatocyte cell types present within the model. This is currently lacking as the cytotoxicity assays routinely used in *in vitro* toxicology research depend on intracellular molecules which are ubiquitously present in all eukaryotic cell types. Parameters commonly used in cytotoxicity assays include the release of the stable cytoplasmic molecule lactate dehydrogenase (LDH) from necrotic cells, or change in the metabolic activity of viable cells, by either relative quantification of intracellular adenosine triphosphate (ATP), or reduction of substrates such as tetrazolium salts by cellular oxidoreductase enzymes using the MTT or MTS colorimetric assays (Lappalainen *et al*., 1994; Mosmann, 1983; Slater, 2001). These intracellular molecules and enzymes that are used in these assays are not cell-specific, and therefore the parameters described above to measure toxicity and cell viability can only be applied accurately in models incorporating homogeneous cultures. Therefore, in the emerging models of DILI which take the field beyond assaying simple single hepatocytes or HLCs, current toxicological endpoints largely reflect the global toxicity of the different cell types in the culture, and are not able to specifically measure only the perturbation of the hepatocytes or HLCs.

In this work, we explore the concept of using microRNA-122 (miR-122) as a potential hepatocyte-enriched marker of toxicity. MicroRNAs (miRNAs) are highly conserved small non-coding RNAs responsible for translational regulation of messenger RNAs (Chen and Rajewsky, 2007; Lagos-Quintana *et al.*, 2002). Some miR species demonstrate high tissue enrichment, with miR-122 shown to be highly enriched and abundant in adult and foetal liver, constituting more than 70% of the total liver miR content (Chang *et al.*, 2004; Lagos-Quintana *et al.*, 2002; Liu *et al.,* 2009; Sempere *et al.,* 2004). miR-122 is involved in hepatic differentiation via a feedback loop with the liver-enriched transcription factor network (Laudadio *et al.*, 2012), and is also highly upregulated in human embryonic stem cell (hESC)-derived hepatocytes compared with undifferentiated stem cells and early endodermal lineage cells (Kim *et al.*, 2011). For these reasons, several studies have evaluated miR-122 in the plasma as a circulating hepatocyte-specific biomarker of various liver injuries, including DILI in rodents and humans caused by drugs such as acetaminophen and heparin (Antoine *et al.*, 2013; Harrill *et al.*, 2012; Starkey Lewis *et al.,* 2011; Wang *et al.*, 2009). Therefore, miR-122 could potentially be used as a bridging biomarker to translate findings from *in vitro* experiments to *in vivo* experiments and the clinical setting. However, to date, the utility of miR-122 as an *in vitro* hepatocyte-enriched marker of drug-induced toxicity has not been explored.

Therefore, using the prototypical hepatotoxicants acetaminophen and diclofenac, we investigated the potential application of miR-122 as a hepatocyte-enriched biomarker of drug-induced toxicity in human primary hepatocytes and HLCs—hepatic models with high levels of intracellular miR-122, and assessed the sensitivity of the miR-122 toxicity assay in comparison with conventional cytotoxicity assays in detecting drug-induced hepatocyte perturbation.

## MATERIALS AND METHODS

### 

#### Human Subjects and Tissue

#### 

Human liver resections from surgical waste tissue were obtained from adult patients (females [*n* = 2], males [*n* = 4]; mean age: 68 [54–76]) undergoing liver resections for hepatocellular carcinoma (*n* = 4) or colorectal cancer metastases (*n* = 2), with full informed consent and ethical approval from the relevant authorities (National Research Ethics Service REC reference: 11/NW/0327).

#### Human Primary Hepatocyte Isolation and Culture

#### 

Human primary hepatocytes were isolated using a previously described method with minor modifications (LeCluyse *et al.*, 2005). Briefly, liver resections were received as surgical waste tissue immediately post-resection (Aintree University Hospital, Liverpool, UK) and transferred on ice in HEPES buffer (10mM HEPES, 136mM NaCl, 5mM KCl, 0.5% [wt/vol] glucose, pH 7.6) to the laboratory. The liver resection specimens were then perfused with HEPES-buffered saline (HBS) followed by digestion with collagenase A (Roche) or collagenase IV (Sigma-Aldrich) in HBS containing calcium. The suspension containing isolated hepatocytes was then filtered through a nylon gauze and purified by centrifugation twice at 80 × g for 5 min at 4°C, before the pellet was resuspended in Williams E medium (Sigma-Aldrich). Following this, cell viability was determined using the trypan blue exclusion method and the isolated hepatocytes were deemed suitable for use in this study only if the viability is more than or equal to 80%. The mean viability of the donor hepatocytes used in this study was 91(84–96)%.

The hepatocytes were then seeded onto collagen-I-coated 24-well plates (BD Beckinson) at 2.5  ×  10^5^ cells/cm^2^ and cultured in Williams E medium supplemented with 1% (vol/vol) insulin-transferrin-selenium (from 100 × stock solution, Life Technologies), 2mM l-glutamine (Sigma-Aldrich), 10^−^^7 ^M dexamethasone (Sigma-Aldrich), and 1% (vol/vol) penicillin-streptomycin (Sigma-Aldrich) at 5% CO_2_ and 37°C. After 3 h, non-attached cells were washed away and overlaid with fresh ice-cold medium containing 0.25 mg/ml of Matrigel (BD Beckinson). The media was replaced the next day at the start of the experiments.

#### Human Cancer Cell Line Culture

#### 

The human hepatoma cell line (HepG2) and the pancreatic cancer cell line (Suit-2) were cultured in Dulbecco’s Modified Eagle’s Medium (DMEM) supplemented with 2mM l-glutamine (Sigma-Aldrich), 10% (vol/vol) fetal bovine serum (FBS; Life Technologies) and 1% (vol/vol) penicillin-streptomycin (Sigma-Aldrich) at 5% CO_2_ and 37°C.

#### Human Pluripotent Stem Cell Culture

#### 

The hESC line HUES7 was maintained on mitotically inactivated murine embryonic fibroblasts (MEFs) as previously reported (Baxter *et al.*, 2009), in KnockOut DMEM (KO-DMEM; Life Technologies) supplemented with 20% (vol/vol) KnockOut Serum Replacement (KOSR; Life Technologies), 0.1mM non-essential amino acids (NEAAs), 2mM l-glutamine (Life Technologies), 1% (vol/vol) penicillin-streptomycin (Life Technologies), 1% (vol/vol) insulin-transferrin-selenium (from 100 × stock, ITS; Life Technologies), 0.1mM beta-mercaptoethanol (Life Technologies), and 4 ng/ml fibroblast growth factor 2 (FGF2; PeproTech).

The hESC line Shef-3 was maintained on embryonic stem cell-qualified Matrigel (BD Beckinson)-coated plates as previously reported (Bone *et al*., 2011), in mTeSR1 (STEMCELL Technologies).

Cell suspensions of human-induced pluripotent stem cells, ChiPSC-18 (DEF-hiPSC, Cellartis by Takara Bio Europe AB), were plated at a density of 70 000 cells/cm^2^ onto a proprietary matrix as per protocol, and maintained using a proprietary feeder-free and defined culture system, DEF-CS 500 (Cellartis by Takara Bio Europe AB). ChiPSC-18 was generated using polycistronic retrovirus technology based on the transduction of the transcription factors Oct3/4, Sox2, Klf4, and c-Myc (Takahashi *et al.,* 2007).

#### Differentiation of Human Pluripotent Stem Cells into HLCs

#### 

The differentiation of HUES7 hESCs toward definitive endoderm was commenced 3–4 days post-passage onto fresh MEFs in Roswell Park Memorial Institute (RPMI) media (Sigma-Aldrich) supplemented with 0.5% (vol/vol) FBS (Life Technologies), 100 ng/ml activin A (AA, PeproTech), and 25 ng/ml Wnt-3a (R&D Systems) for 48 h, followed by 0.5% (vol/vol) FBS (Life Technologies) and 100 ng/ml AA (PeproTech) without Wnt-3a for a further 48 h. Hepatic specification was then carried out for 6 days in Hepatocyte Culture Medium (HCM; Lonza) supplemented with 20 ng/ml bone morphogenetic protein 2 (R&D Systems) and 30 ng/ml FGF2 (PeproTech). For the hepatocyte maturation stage, the HLCs were cultured in HCM supplemented with 20 ng/ml hepatocyte growth factor (HGF, PeproTech) for 5 days followed by HCM supplemented with 10 ng/ml oncostatin M (R&D Systems) and 10^−^^7^ M dexamethasone (Sigma-Aldrich) for a further 15 days.

The differentiation of Shef-3 hESCs toward HLCs was performed using a 3-stage differentiation protocol. Briefly, to induce differentiation to definitive endoderm (DE), Shef-3 hESCs were cultured in RPMI media (Life Technologies) containing 100 ng/ml AA (PeproTech) and 2 μM 1 m for 24 h, followed by 100 ng/ml AA and 0.2% (vol/vol) HyClone FBS (Fisher Scientific) for 48 h. DE cells were then passaged as single cells with Accutase (STEMCELL Technologies) onto Matrigel (BD Beckinson)-coated 96-well plates at a density of 18 000 cells/well. Hepatic specification was carried out for 7 days in KO-DMEM supplemented with l-glutamine (Life Technologies), 0.5% (vol/vol) penicillin-streptomycin (Life Technologies), 1mM NEAA (Life Technologies), 2% (vol/vol) KnockOut Serum Replacement (Life Technologies), 10 ng/ml HGF (PeproTech), and 10 ng/ml fibroblast growth factor 4 (FGF4; PeproTech). To allow for maturation, HLCs were cultured for 14 days in Williams E medium supplemented with l-glutamine (Life Technologies), 0.5% (vol/vol) penicillin-streptomycin, 1% (vol/vol) ITS (from 100 × stock, Life Technologies), 10 ng/ml oncostatin M (PeproTech), 10 ng/ml HGF (PeproTech), 10 ng/ml FGF4 (PeproTech), 10 ng/ml epidermal growth factor (PeproTech), and 10^−^^7^ M dexamethasone (Sigma-Aldrich). During hepatic induction and maturation, medium was replenished every other day.

HLCs generated from ChiPSC-18 were received on Day 23 of differentiation in plated 96-well plate formats (Enhanced hiPS-HEP, Cellartis by Takara Bio Europe AB), and were maintained with proprietary media, HEP-104-SUP (Cellartis by Takara Bio Europe AB).

#### Cytotoxicity Assays

#### 

Human primary hepatocytes, HLCs, and undifferentiated human pluripotent stem cells were incubated for up to 24 h with acetaminophen (0–30mM, Sigma-Aldrich) and diclofenac (0–1mM, Sigma-Aldrich), which were dissolved and diluted to the final test concentrations in the appropriate culture media. The range of concentrations used in the cytotoxicity assays was chosen to incorporate the concentration of 30 times the reported efficacious concentration (*C*_max_) for each compound in humans, which was suggested to be the optimal drug concentration for *in vitro* prediction of human toxicity (O’Brien *et al.*, 2006).

The human primary hepatocytes and the hiPSCs (ChiPSC-18) were treated with the test compounds 24 h after seeding, while for the HLCs generated from ChiPSC-18, cytotoxicity assays were started on Day 31 of differentiation.

##### LDH activity assay

For the human primary hepatocyte samples, both the cellular lysates and media at the end of the experiments were collected and assayed separately for LDH using the Cytotoxicity Detection Kit (Roche). Briefly, the media were first collected and the cells lysed in medium containing 2% (vol/vol) Triton X-100 (Sigma-Aldrich). Both the media and cellular lysate were then stored in −80°C immediately. The level of LDH activity (a surrogate for the quantity of LDH molecules) in each sample was determined separately as per the manufacturer’s instructions and the percentage of LDH activity in the media was expressed as a percentage of the combined total intracellular and extracellular LDH activity in the well.

##### Intracellular ATP assay

The quantification of intracellular ATP in HLCs and undifferentiated human pluripotent stem cells was performed using the CellTiter-Glo Luminescent Cell Viability Assay (Promega). Following dosing of the cells with acetaminophen and diclofenac for 24 h, the media was collected first for miR-122 analysis and the remaining adherent cells used for ATP measurement. Briefly, 50 μl of Dulbecco’s phosphate-buffered saline (DPBS) (Life Technologies) was added to each well followed by 50 μl of CellTiter-Glo reagent which was prepared according to the manufacturer’s protocol. The contents were mixed in a plate shaker for 2 min to induce cell lysis and then incubated for 10 min at room temperature. The cellular lysate was then transferred to an opaque 96-well plate and the luminescent signal measured using a Varioskan Flash spectral scanning multimode reader (Thermo Scientific).

#### Total RNA Extraction

The purification of total RNA containing the miR fraction from media or lysate samples was performed using the miRNeasy mini kit (Qiagen), as per the manufacturer's instructions, with some modifications. Briefly, a typical 40 μl of media or lysate diluted in RNAse-free water up to a total volume of 200 μl was used for RNA purification in each experiment. Following addition of 700 μl of QIAzol (Qiagen) to the diluted media or lysate, and incubation at room temperature for 10 min, 5 μl of a 5 fM solution of cel-lin-4 (Integrated DNA Technologies) was added as a spiked-in exogenous non-human miR to monitor for the efficiency of the miR extraction process by evaluation of the amount of recovered cel-lin-4 in each sample. 140 μl of chloroform was then added and the rest of the protocol was as per the manufacturer’s instruction. The total RNA containing the miR fraction was eluted with RNase-free water and quantification performed using the NanoDrop spectrophotometer (Thermo Scientific). The fully automated platform QIAcube (Qiagen) was used in the extraction of total RNA containing miRs in the HLC media samples.

#### Real-Time Quantitative RT-PCR (qRT-PCR) Analysis of miR-122

MiR-122 levels in each sample were determined using the TaqMan gene expression assay (Applied Biosystems) according to the manufacturer’s protocol. Briefly, a reverse transcription (RT) cocktail mixture containing specific stem-loop RT primers for each target miR species (Applied Biosystems) was prepared as instructed and added to 5 μl of the total RNA elute for complementary DNA (cDNA) synthesis in a total volume of 15 μl. 1.33 μl of cDNA was then mixed with a PCR mixture containing specific stem-loop PCR primers in a total volume of 20 μl. Real-time PCR was then performed in duplicates using the ABI Prism 7000 or ViiA 7 instruments (Applied Biosystems) using a 2-step thermal cycling protocol of 95°C for 10 min followed by 40 cycles of 95°C (15 s) and 60°C (60 s). Ct values were determined using the fluorescent signal produced from the TaqMan probes. The number of copies of miR-122 in each PCR reaction was quantified using the absolute quantification method with a standard curve of cel-lin-4 cDNA used as a surrogate for miR-122 cDNA, due to its similar nucleotide length and to avoid contamination of PCR reactions with synthetic miR-122. The total number of copies of miR-122 in each sample was then extrapolated from this figure. The amplification efficiency of cel-lin-4 cDNA was confirmed in independent experiments to be comparable to that of miR-122 cDNA, with similar standard curves constructed. For the human primary hepatocyte samples, both the total copies of miR-122 in the cellular and media components were determined separately, and the percentage of miR-122 in the media was expressed as a percentage of the combined total of intracellular and extracellular miR-122 copies in the well, analogous to the LDH cytotoxicity assay.

#### Immunocytochemistry

Immunocytochemistry was performed on undifferentiated ChiPSC-18, 1 day after passage, and at Day 28 of differentiation for ChiPSC-18-derived-HLCs, as previously described (Ulvestad *et al.*, 2013). Briefly, cells were fixed in 4% (wt/vol) formaldehyde (Histolab) for 20 min followed by repeated washes in DPBS (Life Technologies). Fixed cells were incubated in a blocking buffer (DPBS, Life technologies) containing 5% (wt/vol) skim milk (Sigma-Aldrich) and 0.2% (vol/vol) Triton X-100 (Sigma-Aldrich) for 30 min followed by overnight incubation with the relevant primary antibody at 4°C. After 3 washes in DPBS (Life Technologies), the fixed cells were incubated with the appropriate secondary antibody for 2 h in the dark, at room temperature, before the cells were washed 3 times again with DPBS containing 0.5 μg/ml 4′,6-diamidino-2-phenylindole (DAPI, Sigma–Aldrich) for nuclear staining, and mounted in Fluorescence Mounting Medium (Dako). Primary and secondary antibodies were diluted in an incubation solution consisting of 1% (vol/vol) bovine serum albumin (Sigma-Aldrich) and 0.2% (vol/vol) TritonX-100 (Sigma-Aldrich). The following primary and secondary antibodies were used: mouse anti-Oct3/4 (1:200, sc-5279, SantaCruz Biotechnology), rabbit anti-HNF4α (1:400, sc-8987, Santa Cruz Biotechnology), rabbit anti-α-1-antitrypsin (1:200, a0012, Dako), mouse anti-cytokeratin-18 (1:100, M7010, Dako), Alexa Fluor 488 goat anti-mouse IgG (1:1000, A-11029, Life Technologies), Alexa Fluor 488 donkey anti-rabbit IgG (1:1000, A-21206, Life Technologies), and Alexa Fluor 594 goat anti-mouse IgG (1:1000, A-11032, Life Technologies). Stainings were analyzed using a fluorescence microscope (Eclipse TE2000-U, Nikon), a digital camera (DXM1200C, Nikon) and corresponding software (Act-1C software for DXM1200C camera, Nikon). Technical control stainings without primary antibodies were performed for all secondary antibodies, and were all negative (data not shown).

#### Statistical analysis

For comparison of the intracellular miR-122 level between models, the mean and the standard error of the mean of each model were determined. For comparisons between each model with human primary hepatocytes, the Mann-Whitney non-parametric test was used, whereas the Dunn’s multiple comparison test was used for comparison of more than 2 models. For the cytotoxicity assays, the unpaired *t*-test was used for pairwise comparisons, while correlation analyses were performed using the Pearson correlation test. For all tests, *P* < 0.05 was considered significant. Statistical analyses were performed using GraphPad Prism 6 (GraphPad Software, La Jolla, California).

## RESULTS

### 

#### Intracellular miR-122 Levels Reflect the Physiological Relevance of Human Hepatic Models

Freshly isolated human primary hepatocytes are currently considered to be the most physiologically relevant single cell *in vitro* hepatic model, particularly due to their functional recapitulation of the *in vivo* metabolic processes such as phase I and II enzyme activities, glucose metabolism, and ammonia detoxification (Godoy *et al.*, 2013; Kia *et al*., 2013). Therefore, to investigate the relevance of miR-122 as a potential biomarker in *in vitro* hepatic models, we first performed quantification of the intracellular level of miR-122 among the commonly used hepatic models of HepG2 (a hepatocellular cancer cell line) and human pluripotent stem cell-derived HLCs in direct comparison to freshly isolated and plated human primary hepatocytes from adult donors.

**FIG. 1.**
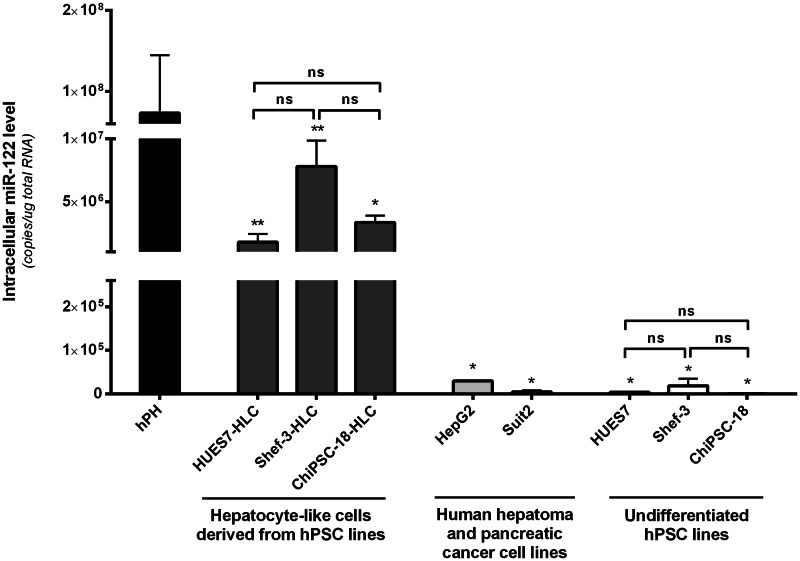
Quantitative comparison of intracellular miR-122 between human hepatic models. Data are presented as the mean total number of copies of intracellular miR-122 per µg of total RNA ± SEM for human primary hepatocytes, hPH (*n* = 6 different donors), HLCs differentiated from HUES7 and Shef-3 hESC lines (*n* = 3 separate differentiation experiments each), HLCs differentiated from ChiPSC-18 hiPSC line (*n* = 3 separate differentiation experiments), HepG2 (*n* = 3 biological replicates from one experiment), Suit2 (*n* = 3 biological replicates from one experiment) and undifferentiated human pluripotent stem cell (hPSC) lines (*n* = 3 independent experiments each). The Mann-Whitney non-parametric test was used to compare each model against human primary hepatocytes for statistical significance, whereas the Dunn’s multiple comparison test was used when performing comparisons between HLCs and between the undifferentiated pluripotent stem cell lines. *P < 0.05, **P < 0.01, ns denotes non-significance.

We found that the mean intracellular miR-122 level normalized to the amount of total RNA used for qRT-PCR is the highest in human primary hepatocytes with a 9- to 41-fold lower expression in hPSC-derived HLCs, and 2500-fold lower in HepG2 (Fig. 1). Human primary hepatocytes also expressed significantly more miR-122 compared with undifferentiated hPSCs (3900- to 78 000-fold lower) and the pancreatic cancer cell line Suit2 (17 000-fold lower), which was included as part of the comparison as a negative control for non-hepatic cells of the endodermal lineage.

Overall, the results in Figure 1 confirm that miR-122 is highly enriched in human primary hepatocytes and provide a quantitative comparison of intracellular miR-122 among the most relevant human hepatic *in vitro* models currently used.

#### MiR-122 Expression Increases during Directed Differentiation of hESCs and hiPSCs Toward HLCs

In HLCs differentiated from their respective hESC lines, the mean fold increase of miR-122 from basal levels in their originating undifferentiated hESC lines was similar (420- and 430-fold change, respectively), whereas in the HLCs differentiated from the hiPSC line (ChiPSC-18), the mean increase in miR-122 level was higher at 3500-fold (Fig. 1). However, there was no significant difference between the mean intracellular miR-122 levels among the HLCs derived from the 2 hESC lines and ChiPSC-18, or between the low levels of mean intracellular miR-122 detected in the undifferentiated pluripotent stem cell lines.

#### The Relative Level of miR-122 Detected in the Media Correlates with the Extracellular Release of LDH in Drug-Induced Toxicity of Human Primary Hepatocytes

To explore the potential of miR-122 as an *in vitro* marker of drug-induced cellular perturbation, we first compared its performance against the conventional marker of cellular necrosis, LDH, using human primary hepatocytes. We chose to validate miR-122 against LDH, as the levels of LDH and miR-122 can be readily measured in both the cellular lysate and media component, which allows for direct comparison of the sensitivity of both markers in detecting drug-induced toxicity.

**FIG. 2.**
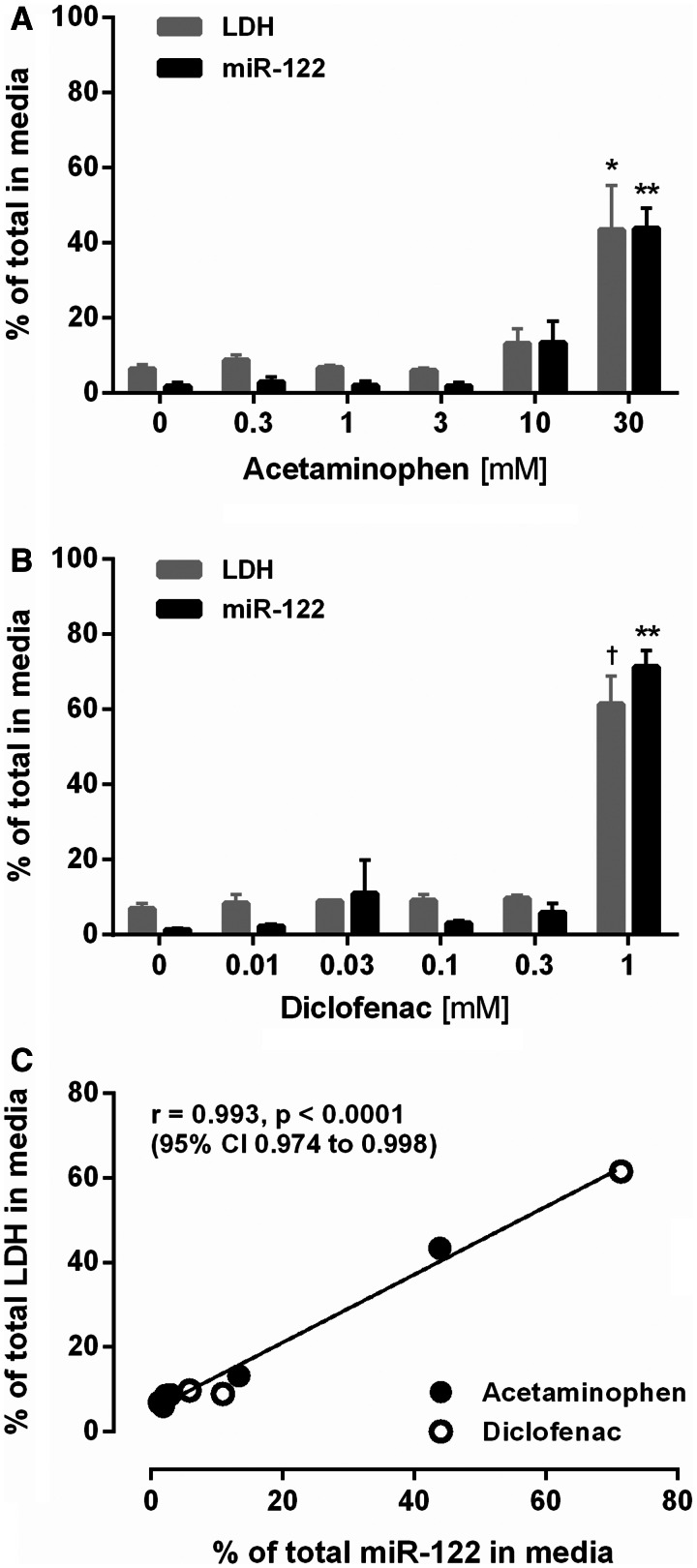
Dose-response of human primary hepatocytes after treatment with acetaminophen and diclofenac. Percentage of total LDH and miR-122 in the media of human primary hepatocytes at 24 h post-treatment with (A) acetaminophen and (B) diclofenac. Data are presented as the mean ± SEM from 3 independent experiments using different donors of human primary hepatocytes. For each donor batch of hepatocytes, parallel experiments were performed in duplicates to compare the sensitivity of LDH and miR-122 as biomarkers. The unpaired *t*-test was used to compare the statistical significance of the dose-response as measured by the percentage of total marker in the media for each concentration of hepatotoxicant against the respective untreated controls. *P < 0.05, **P < 0.01, ^†^*P* < 0.0001. (C) Correlation of the percentage of total LDH in the media against the percentage of total miR-122 in the media, using paired mean values for each condition obtained from experimental results summarized in Figures 2A and 2B. *r* denotes Pearson’s correlation coefficient, CI denotes the confidence interval of *r*.

Dose-response experiments using human primary hepatocytes incubated with the known hepatotoxicants acetaminophen and diclofenac for 24 h (Figs. 2A and 2B) were performed. The LDH activity in the media and cellular fraction of each well were separately measured to calculate the relative percentage of total LDH activity in the media. In parallel experiments, using the same donor-batch of human primary hepatocytes, we also quantified the number of copies of miR-122 in the media and cellular fraction in response to acetaminophen and diclofenac, and calculated the relative percentage of total miR-122 in the media (Figs. 2A and 2B). We found that the level of miR-122 increased in the media with increasing concentrations of acetaminophen and diclofenac, which paralleled the extracellular release of intracellular LDH. Significant levels of both molecules were detected in the media at the maximal concentrations of acetaminophen and diclofenac used in this study (Figs. 2A and 2B). By plotting the paired values of each biomarker obtained from the experiments using various concentrations of acetaminophen and diclofenac in a scatter graph, we confirmed that there was a high positive correlation between the levels of LDH and miR-122 in the media (Pearson’s correlation coefficient, *r* = 0.993, *P* < 0.0001, 95% CI 0.974–0.998) (Fig. 2C). This suggests that using the model of human primary hepatocytes, miR-122 is equivalent to LDH when used as an *in vitro* biomarker of drug-induced toxicity.

**FIG. 3.**
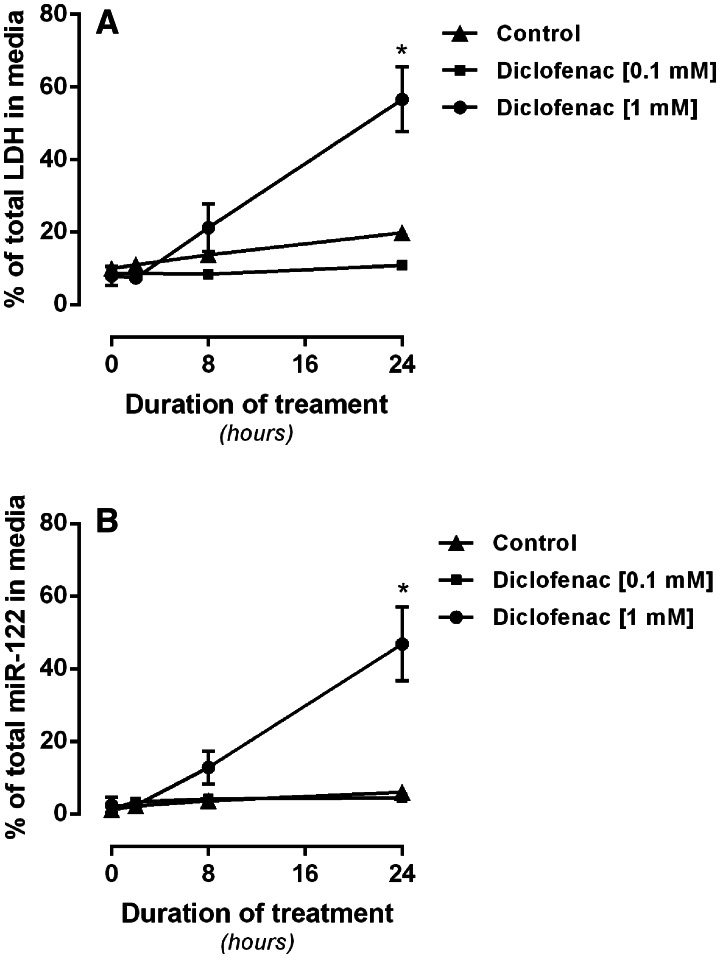
Hepatotoxicity of human primary hepatocytes treated with diclofenac up to 24 h. Time-course experiment of human primary hepatocytes treated with diclofenac up to 24 h as measured by (A) percentage of total LDH in the media and (B) percentage of total miR-122 in the media. Data are presented as the mean ± SEM from 3 independent experiments using different donors of human primary hepatocytes. For each donor batch of hepatocytes, parallel experiments were performed in duplicates to compare the sensitivity of LDH and miR-122 as biomarkers. The unpaired *t* test was used to compare the statistical significance of the percentage of total marker in the media at each time-point compared with untreated controls. *P < 0.05.

We also performed a time-course experiment on the hepatocytes treated with diclofenac over 24 h and observed comparable trends in both biomarkers in the media with a concentration of 1mM (Figs. 3A and 3B). Increased levels of both biomarkers were detected in the media after 2 h, although significant levels were only detected at 24 h using either biomarker (Figs. 3A and 3B). This suggests miR-122 is as sensitive as LDH when used as a toxicity marker for human primary hepatocytes but the time-course experiments using diclofenac as the test compound did not point toward the possibility of miR-122 providing an earlier signal of hepatocyte perturbation compared with LDH.

We also observed that the miR-122 level in the media has a wider dynamic range compared with LDH. In both sets of dose-response experiments, the untreated human primary hepatocytes showed a baseline mean percentage of total LDH in the media at 24 h of 6.4% and 6.9%, respectively, compared with a baseline mean percentage of total miR-122 of 1.8% and 1.4% (Figs. 2A and 2B). When a toxic concentration of 30mM acetaminophen was used, the mean increase over baseline of LDH in the media was 6.8-fold compared with a higher mean increase of 25-fold for miR-122 (Fig. 2A). Similarly, human primary hepatocytes treated with 1mM diclofenac showed a mean increase over baseline of LDH in the media of only 8.9-fold compared with a higher mean increase of 53-fold for miR-122 (Fig. 2B).

We then sought to simplify the miR-122 assay further and observed that the estimation of the total copies of miR-122 in the media alone is sufficiently predictive of the relative percentage of total miR-122 in the media. It has a wide dynamic range and the denominator of the total number of copies of miR-122 in both the cellular lysates and the media component remained fairly constant throughout the various validation experiments (Supplementary Figs. 1 and 2). Using the experimental values obtained from the dose-response experiments of hepatocytes treated with various concentrations of acetaminophen and diclofenac, we found a high positive correlation between the mean percentage of total miR-122 and the mean number of copies of miR-122 in the media (Pearson’s correlation coefficient, *r* = 0.967, *P* < 0.0001, 95% CI 0.882–0.991) (Supplementary Fig. 3A). There was also a similarly high positive correlation between the mean percentage of total LDH activity and the mean number of copies of miR-122 in the media (Pearson’s correlation coefficient, *r* = 0.945, *P* < 0.0001, 95% CI 0.810–0.985) (Supplementary Fig. 3B). Therefore, by absolute quantification of the number of copies of miR-122 in the media alone, the miR-122 assay as a biomarker of toxicity is made simpler without the need to measure the miR-122 level in the cell lysate component. Drug-induced perturbation of the hepatic models can be evaluated using only the media component by relative comparison with the baseline levels of miR-122 in the media.

#### The Level of miR-122 in the Media Reflects Hepatocyte-Specific Drug-Induced Toxicity in hiPSC-Derived HLCs

We then explored the use of miR-122 in complex human hepatic models which may not be homogeneous such as cultures of HLCs, where the differentiation efficiency can be variable and not 100% predictable (Baxter *et al.,* 2010; Kia *et al.,* 2013). Conventional cellular markers of toxicity such as LDH and ATP, and others used in multiparametric high content screening platforms, do not discriminate between toxicity affecting hepatocyte and non-hepatocyte cells present in the culture (Rausch, 2006). We hypothesized that the hepatocyte-enriched expression of miR-122 could be used to detect selectively drug-induced perturbation of the HLCs in non-homogeneous cell cultures.

**FIG. 4.**
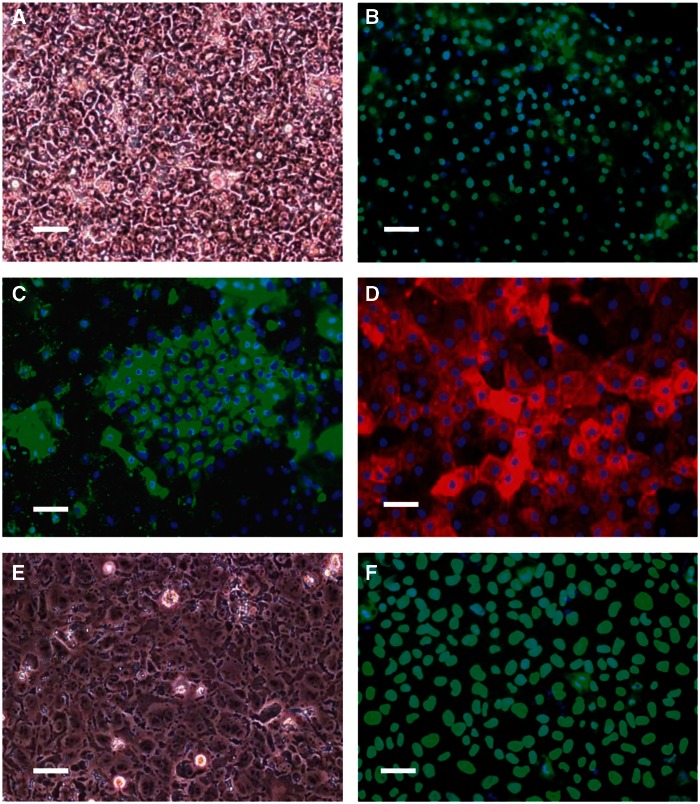
Characterization of ChiPSC-18-derived hepatocyte-like cells (HLCs) and undifferentiated human induced pluripotent stem cells (ChiPSC-18). Phase contrast pictures of ChiPSC-18-derived HLCs (A) and undifferentiated ChiPSC-18 (E). Immunocytochemical analysis of the expression of the hepatic markers HNF4α (B), α-1-antitrypsin (C), and cytokeratin-18 (D) in combination with DAPI (nuclear stain) for ChiPSC-18-derived HLCs at day 28 of differentiation, and the expression of the pluripotency marker Oct3/4 in undifferentiated ChiPSC-18 (F). Scale bar: 50 µm (A–F). Hepatocyte nuclear factor 4, HNFα; 4′,6-diamidino-2-phenylindole, DAPI.

The use of miR-122 as an *in vitro* biomarker in HLCs was explored using ChiPSC-18-derived HLCs, which were characterized with immunocytochemical analysis and confirmed to express the hepatic markers hepatocyte nuclear factor 4 alpha (HNF4α), alpha-1-antitrypsin and cytokeratin-18 (Figs. 4A–D), and did not express the pluripotency marker Oct3/4 (data not shown). About 90% of the HLCs have a typical hepatic morphology and HNF4α immuno-positive nuclei. The HLC cultures were incubated with the same hepatotoxicants (acetaminophen and diclofenac), and compared against the sensitivity of intracellular ATP quantification by multiplexing both assays in the same experiments. We chose to use the intracellular ATP assay instead of the LDH activity assay to compare against the miR-122 assay for the cytotoxicity experiments utilizing HLCs, as the much smaller volume of media available for each culture of HLCs (100 µl total volume per well in a 96-well plate format) did not allow for sufficient material for both extraction of miRs and quantification of the LDH activity in the media. However, the intracellular ATP assay has been separately validated by our group for assessment of drug-induced hepatocyte perturbation in freshly isolated adult human primary hepatocytes, using a similar dose range of acetaminophen and diclofenac (*n* = 8 and 6 different donors, respectively). In those experiments, the endpoint of change in intracellular ATP level was confirmed to correlate significantly with the percentage of total LDH activity in the media, percentage of total miR-122 in the media and the number of copies of miR-122 per million cells in the media (data to be published elsewhere).

**FIG. 5.**
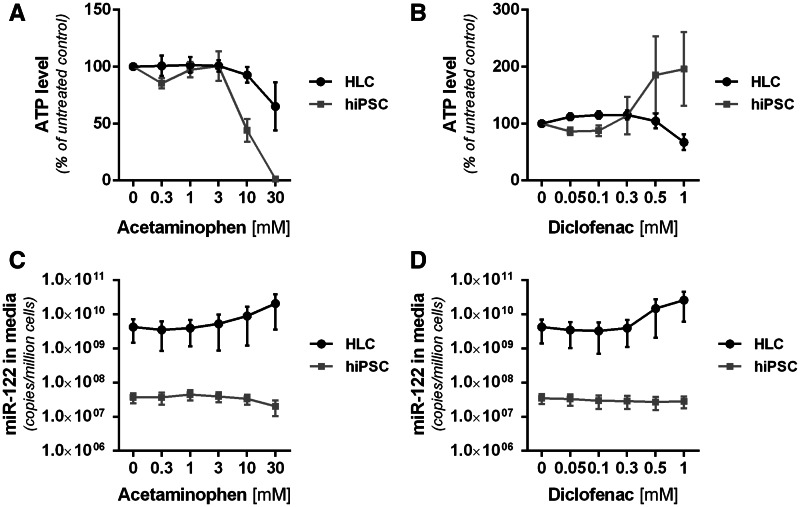
Dose-response of hepatocyte-like cells (ChiPSC-18-derived HLCs) and undifferentiated human-induced pluripotent stem cells (ChiPSC-18) after treatment with acetaminophen and diclofenac. (A, B) Intracellular ATP level (expressed as percentage of vehicle control) and (C, D) number of copies of miR-122 per million cells in the media of hiPSC-derived HLCs and undifferentiated hiPSCs treated with acetaminophen and diclofenac for 24 h. Data are presented as the mean ± SEM from 3 independent experiments. For the HLCs, each independent experiment represented HLCs from separate differentiation experiments, whereas for the hiPSCs, each independent experiment represented separate batches of hiPSCs plated on different days. The cytotoxicity assays were performed in at least two replicates on HLCs and hiPSCs cultured in 96-well plates. The number of copies of miR-122 estimated in the media was normalized to the number of cells estimated to be present in the HLC and hiPSC cultures to allow for a direct comparison of absolute quantities of miR-122 in the media. The density of HLCs present in a single well was estimated to be about 1  ×  10^5^ cells/cm^2^ when the HLC culture was fixed and stained with the nuclear stain DAPI (data not shown). As the total cell culture surface per well was 0.32 cm^2^, a total number of 3.2 × 10^4^ HLCs per well was obtained. For the hiPSCs, a total number of 2.2 × 10^4^ hiPSCs per well was obtained using the plating density of 7 × 10^5^ cells/cm^2^.

In parallel experiments, we also used the undifferentiated hiPSCs (ChiPSC-18) (Figs. 4E and 4F) from which the HLCs were derived from, as a surrogate for poorly differentiated cells present in HLC cultures that do not possess a hepatic phenotype and express a very low level of intracellular miR-122 relative to hepatic cells, to examine the hepatic specificity of miR-122 as an *in vitro* marker of toxicity. Each batch of HLCs or hiPSCs were treated with acetaminophen or diclofenac over 24 h, and the lysate was used for the ATP assay, while the media from the same well was collected in order to estimate the number of copies of miR-122 (Figs. 5A–D). Using the ATP assay, perturbation of the HLCs was detected, with a mean reduction of ATP of 35% and 33%, respectively, at 24 h, when the cells were treated with the highest concentrations of acetaminophen and diclofenac (Figs. 5A and 5B). However, the non-hepatocyte model of hiPSCs displayed a greater reduction in ATP levels, with lower concentrations of acetaminophen (56% with 10mM and 99% with 30mM), confirming ATP as a generic and non-cell-type-specific marker of cellular perturbation (Fig. 5A). In contrast, the hiPSCs, when treated with diclofenac, displayed increased ATP levels of up to 196% of baseline (Fig. 5B).

**FIG. 6.**
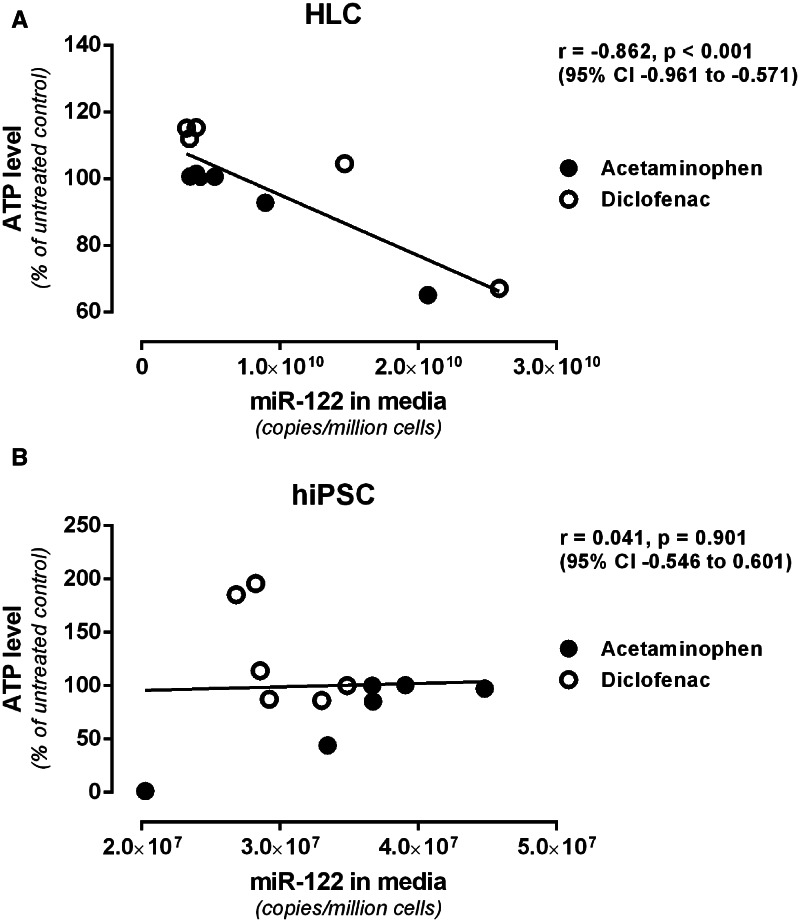
Correlation of intracellular ATP activity with miR-122 level in the media. Correlation between intracellular ATP activity (expressed as percentage of vehicle control) and number of copies of miR-122 per million cells in the media of (A) hiPSC-derived HLCs and (B) undifferentiated hiPSCs, treated with acetaminophen and diclofenac over 24 h using paired mean values obtained from experimental results summarised in Figure 4. *r* denotes Pearson’s correlation coefficient, CI denotes the confidence interval of *r*.

Using miR-122 as a marker for drug-induced toxicity in the same experiments, a trend toward an increase in the number of copies of miR-122 in the media of HLCs was noted with acetaminophen and diclofenac, with a mean 4-fold and 6-fold increase from baseline, respectively, at the highest concentrations used in these experiments (Figs. 5C and 5D). This suggested that when the miR-122 assay was used as a toxicity assay for the HLCs, its performance was as sensitive as the ATP assay. This was confirmed by a correlation analysis using the paired values of both assays from the experiments using HLCs treated with acetaminophen and diclofenac at various concentrations (Fig. 6A). By plotting the values in a scatter graph, a significant negative correlation was found (Pearson’s correlation coefficient, *r* = −0.862, *P* < 0.001, 95% CI −0.961 to −0.571).

However, in contrast to the varying intracellular ATP profile seen in the hiPSCs incubated with acetaminophen and diclofenac (Figs. 5A and 5B), the miR-122 level in the media did not display any increasing trend throughout the range of concentrations of the hepatotoxicants (Figs. 5C and 5D). Analysis utilizing the paired values of both biomarkers from the experiments using hiPSCs described above, also showed no correlation between the levels of miR-122 in the media and the change in intracellular ATP levels in the hiPSCs (Pearson’s correlation coefficient, *r* = 0.041, *P* = 0.901, 95% CI −0.546 to 0.601).

The total number of copies of miR-122 expressed by an equivalent number of hiPSCs was also at least 78-fold less than that expressed by the HLCs, and therefore the potential contributory effect of miR-122 from the non-hepatocyte cells in HLC cultures toward the total number of copies of miR-122 measured in the media was negligible. More importantly, at the toxic concentrations of acetaminophen and diclofenac, where a reduction of intracellular ATP level and an increase in miR-122 in the media of treated HLCs was detected (Figs. 5A–D), the corresponding miR-122 level in the media of hiPSCs treated with the same concentrations remained unchanged. Thus, the source of increased level of miR-122 in a heterogeneous culture such as HLCs treated with toxic concentrations of hepatotoxicants was likely to be from hepatic cells with a high level of intracellular miR-122—in this case well-differentiated HLCs.

## DISCUSSION

MicroRNA-122 has been shown to be a liver-enriched and -abundant miR, which could be useful as a bridging biomarker of drug-induced hepatotoxicity to translate findings from *in vitro* experiments to *in vivo* experiments and the clinical setting.

However, quantitative evaluation of its abundance in various *in vitro* hepatic models currently used for the study of drug-induced hepatotoxicity in comparison to human primary hepatocytes has not been considered. For this study, we first confirmed that the *in vitro* hepatic models examined express high levels of miR-122, in contrast to the non-hepatic models (Fig. 1). The lower intracellular miR-122 levels in the various models in comparison to freshly isolated human primary hepatocytes suggest that the quantity of intracellular miR-122 broadly reflects the models’ degree of hepatic phenotype. Although HLCs differentiated in conventional 2D cultures have not yet been reported to display a full complement of drug metabolizing enzymes at comparable levels to human primary hepatocytes (Kia *et al.*, 2013; Sjogren *et al.*, 2014; Ulvestad *et al.*, 2013), nevertheless HLCs derived from pluripotent stem cell lines with normal genotypes would be expected to exhibit a physiologically more relevant hepatic phenotype compared with liver cancer cell lines. This has been confirmed in studies directly comparing the drug metabolizing capacity of pluripotent stem cell-derived HLCs and the hepatocellular cancer cell lines, HepG2 and HuH7 (Brolen *et al.,* 2010; Sjogren *et al.*, 2014; Soderdahl *et al.,* 2007). Therefore, miR-122 can also be potentially exploited as a biomarker of physiological relevance of current and emerging *in vitro* hepatic models for mechanistic studies of DILI, by correlating their intracellular level of miR-122 to the biochemical and functional phenotype. This work is underway and will be reported elsewhere.

Figure 1 also supports a previous observation that the miR-122 expression increases during directed differentiation of hESCs toward HLCs (Kim *et al.,* 2011). In that study, only a single hESC line was used for HLC generation using a different protocol to those used in this study. Hence, our study adds to that finding as we used 2 different hESC lines, maintained in both feeder and feeder-free culture conditions, and differentiated toward HLCs using 2 disparate protocols. This study also demonstrates for the first time that HLCs differentiated from hiPSCs exhibit a similar trend of increase in miR-122 expression to hESC-derived HLCs, although only one hiPSC line was examined in this study.

In homogeneous hepatic models, such as plated human primary hepatocytes or HLC cultures derived with high differentiation efficiencies, we propose that the miR-122 cytotoxicity assay, which has marked concordance with the conventional assays utilizing generic markers (Figs. 2, 3, and 6), is a more relevant cell-type-specific assay to examine drug-induced toxicity of the hepatic cells. One major advantage of using miR-122 as an *in vitro* biomarker is the potential to translate such *in vitro* findings *to in vivo* animal models and human samples using the same assay, as miR-122 in the plasma has been shown to be an appropriate marker of acetaminophen-induced liver injury in a mouse model and in humans (Antoine et al., 2013; Harrill et al., 2012; Starkey Lewis *et al.*, 2011; Wang *et al.*, 2009). In other words, miR-122 can be used to bridge results in *in vitro* experiments to clinical findings, and conversely used to link findings from clinical studies to inform on the relevance of *in vitro* models being developed for the study of DILI, which the current conventional *in vitro* markers such as LDH and ATP could not provide.

Similarly, in models where heterogeneity of the hepatic phenotype is present such as in HLC cultures with moderate or variable differentiation efficiencies, the miR-122 cytotoxicity assay may be essential as it allows for specific assessment of the effect of hepatotoxicants on the hepatic cells and the ability to discriminate from the “noise” of the surrounding non-hepatocytes. For example, determining the relative intracellular ATP levels between mature HLCs and poorly differentiated endodermal progenitors or differentiated cells of other lineages in a typical HLC culture would not be possible, as the ATP assay readout reflects the composite of all the ATP content present within a culture. Therefore, it is not possible to measure specifically the dose-response of mature HLCs toward hepatotoxicants in a heterogeneous culture using the ATP assay or any other assays utilizing biomarkers ubiquitously present in all cells, such as LDH. This was borne out in our study where the non-hepatocyte model of hiPSCs displayed higher levels of toxicity as measured by the reduction of intracellular ATP levels at lower concentrations of acetaminophen, likely through non-CYP-dependent mechanisms, confirming ATP as a generic and non-cell-type-specific marker of cellular perturbation (Fig. 5A). Conversely, the ATP levels in hiPSCs treated with diclofenac increased up to 196% of baseline (Fig. 5B), though the reason for this increase is unknown and the effect of diclofenac on human pluripotent stem cells has not been reported in the literature.

The utility of the miR-122 cytotoxicity assay for the specific assessment of the effect of hepatotoxicants on hepatic cells is also potentially applicable in complex 2D or 3D hepatic models of human hepatocytes cocultured with their non-parenchymal counterparts, which are widely considered to be the ideal *in vitro* hepatic models as they mimic the complex multicellular interactions that recapitulate the niche environment in the human liver (Godoy *et al*., 2013). Indeed, progress has been reported in using human hepatocytes and pluripotent stem cell-derived HLCs in cocultures with non-hepatocyte cells to produce *in vitro* hepatic models with an improved functional phenotype (Bhatia *et al.*, 1999; Takebe *et al.*, 2013). Although the coculture with non-hepatocyte cells enhances the functional phenotype of the hepatic cells, their concomitant presence could conceivably complicate the analysis of toxicity specifically targeting the hepatocytes, using the currently available conventional cytotoxicity assays. Indeed, one reported approach in a coculture model of rat primary hepatocytes and murine stromal support cells to calculate the hepatocyte-only responses for non-hepatocyte-specific endpoints such as intracellular ATP and glutathione in response to drug-induced toxicity, was to perform simultaneous assays on cultures of the stromal cells alone and to subtract subsequent values from that obtained from the cocultures (Ukairo *et al.*, 2013). However, this approach does not truly encompass the effect of the multicellular interaction between the hepatocytes and the stromal cells in response to events causing cellular perturbation, such as drug-induced toxicity. An ideal biomarker in these cocultures would be one which is specifically and dynamically changed only in the hepatic cells, and can be measured directly in the cocultures. We propose that miR-122 is one such hepatocyte-enriched marker that can be applied in hepatic models that incorporate hepatocytes or HLCs that express high levels of miR-122.

Although we have shown the utility of miR-122 in the human primary hepatocyte and HLC models, validation experiments of its predicted utility in complex coculture hepatic models still need to be conducted. Similarly, the utility of the miR-122 assay could be considered in high content cellular screening assays widely adopted by the pharmaceutical industry, which currently look at organelle-specific toxicity or screen for cellular perturbation events that are not hepatocyte specific (Rausch, 2006). Therefore, in these screening assays changes in endpoints will be detected as readily in non-hepatocyte cells as in hepatocytes.

In a practical sense, as the miR-122 cytotoxicity assay described here uses quantitative RT-PCR, only a small amount of miR-122-containing media is required—we have extracted adequate miR-122 for analysis using as little as 50 μl of media from a total volume of 100 μl used to maintain 2.2  ×  10^4^ hiPSCs in a 96-well format. This assay can also be multiplexed simultaneously with other cytotoxicity assays that require a portion of the media and/or cellular lysate components. Furthermore, the miR-122 assay also allows for repetitive sampling during chronic dosing experiments, which may be a more relevant approach to studying human DILI in *in vitro* hepatic models, and will also enable sampling of hepatocyte damage in complex 3D bioreactor cultures. A disadvantage of this assay compared with other conventional cytotoxicity assays that mainly use plate readers for endpoint readouts is that the quantification of miR-122 in the media for each sample involves multiple preprocessing steps of RNA extraction and RT. So far, the miR-122 cytotoxicity assay has not been applied as high-throughput readouts, but our workflow in using this assay benefits from the availability of automated platforms of RNA extraction, an automated instrument for PCR setup and a PCR platform for analyzing a 384-well PCR plate format.

Although the application of miR-122 for detecting drug-induced toxicity is shown in this study, the basis for the increase of miR-122 in the media during drug-induced toxicity is still unclear. However, our finding of a high correlation between the increases in LDH and miR-122 in the media of human primary hepatocytes treated with hepatotoxicants suggests that miR-122 may be passively released from necrotic cells (Figs. 2 and 3), which is in keeping with the finding that miR-122 predominantly is increased in the protein-rich fraction rather than the exosome-rich fraction in the plasma/serum samples of a mouse model of acetaminophen-induced liver injury (Bala *et al.*, 2012). It was postulated that as acetaminophen-induced liver injury is severe and rapid, miRs may be released primarily through leakage from dying hepatocytes, as opposed to release through exosomes in liver injuries which are less severe and slower such as in alcoholic liver disease. However, increased cellular-mediated release of miR-122 in microparticles, exosomes, or protein complexes as a response to toxic chemical exposure cannot be excluded (Salminen *et al.*, 2011). It is also unclear if drug-induced toxicity *per se* or individual drugs have any effect on the synthesis or degradation of mature miR-122, although data shown in Supplementary Figures 1 and 2 suggest that the hepatotoxicants examined in this study at least, have no effect on the steady state level or the total number of copies of miR-122 in the human primary hepatocyte cultures. Defining the effects of other hepatotoxicants that induce DILI through other mechanisms such as cholestasis and steatosis on the dynamics of miR-122 release may help in unravelling this uncertainty. Nevertheless, the data presented here establish the potential of miR-122 as a useful *in vitro* marker of drug-induced toxicity. Bridging *in vitro* and *in vivo* studies can now be performed to further define the mechanism(s) of miR-122 release, which will enhance the mechanism-based utility of miR-122 both as a quantitative and qualitative marker of liver injury.

In summary, this report demonstrates that miR-122 detection in cell culture media can be used as an *in vitro* marker of drug-induced cytotoxicity in homogeneous cultures of hepatic cells, and also can be applied as a hepatocyte-enriched marker of toxicity in heterogeneous cultures of hepatic cells. Furthermore, these results indicate the potential of miR-122 to be used in hepatic models using coculture with non-hepatocyte cells, where use of conventional cytotoxicity assays employing generic cellular markers may not be appropriate. We show that the sensitivity of the miR-122 cytotoxicity assay is similar to conventional assays measuring LDH activity and intracellular ATP levels in hepatic cultures, and that this can be multiplexed with other assays. Future challenges include defining the mechanism(s) by which miR-122 is released into the media and understanding the effect of drugs on miR-122 biogenesis and stability.

## SUPPLEMENTARY DATA

Supplementary data are available online at http://toxsci.oxfordjournals.org/.

## FUNDING

Funded by the Stem Cells for Safer Medicines (SC4SM) and supported by the Medical Research Council (MRC) Centre for Drug Safety Science (grant number G0700654). R.K. is a Medical Research Council (MRC) Clinical Training Fellow supported by the North West England MRC Fellowship Scheme in Clinical Pharmacology and Therapeutics, which is funded by the Medical Research Council (grant number G1000417/94909), ICON, GlaxoSmithKline, AstraZeneca, and the Medical Evaluation Unit. R.L.C.S-Y. is funded by the Innovative Medicines Initiative MIP-DILI programme (grant agreement number 115336). J.A.H. is jointly funded by the Biotechnology and Biological Sciences Research Council of UK and AstraZeneca. N.A.H. is funded by the Wellcome Trust.

## Supplementary Material

Supplementary Data

## References

[kfu269-B1] Antoine D. J. Dear J. W. Lewis P. S. Platt V. Coyle J. Masson M. Thanacoody R. H. Gray A. J. Webb D. J. Moggs J. G. (2013). Mechanistic biomarkers provide early and sensitive detection of acetaminophen-induced acute liver injury at first presentation to hospital. Hepatology 58**,** 777–787. 23390034 10.1002/hep.26294 PMC3842113

[kfu269-B2] Bader A. Knop E. Kern A. Boker K. Fruhauf N. Crome O. Esselmann H. Pape C. Kempka G. Sewing K. F. (1996). 3-D coculture of hepatic sinusoidal cells with primary hepatocytes-design of an organotypical model. Exp. Cell. Res. 226**,** 223–233. 8660959 10.1006/excr.1996.0222

[kfu269-B3] Bala S. Petrasek J. Mundkur S. Catalano D. Levin I. Ward J. Alao H. Kodys K. Szabo G. (2012). Circulating microRNAs in exosomes indicate hepatocyte injury and inflammation in alcoholic, drug-induced, and inflammatory liver diseases. Hepatology 56**,** 1946–1957. 22684891 10.1002/hep.25873 PMC3486954

[kfu269-B4] Baxter M. A. Camarasa M. V. Bates N. Small F. Murray P. Edgar D. Kimber S. J. (2009). Analysis of the distinct functions of growth factors and tissue culture substrates necessary for the long-term self-renewal of human embryonic stem cell lines. Stem Cell Res. 3**,** 28–38. 19428319 10.1016/j.scr.2009.03.002

[kfu269-B5] Baxter M. A. Rowe C. Alder J. Harrison S. Hanley K. P. Park B. K. Kitteringham N. R. Goldring C. E. Hanley N. A. (2010). Generating hepatic cell lineages from pluripotent stem cells for drug toxicity screening. Stem Cell Res. 5**,** 4–22. 20483202 10.1016/j.scr.2010.02.002 PMC3556810

[kfu269-B6] Bhatia S. N. Balis U. J. Yarmush M. L. Toner M. (1999). Effect of cell–cell interactions in preservation of cellular phenotype: cocultivation of hepatocytes and nonparenchymal cells. FASEB J. 13**,** 1883–1900. 10544172 10.1096/fasebj.13.14.1883

[kfu269-B7] Bock C. Kiskinis E. Verstappen G. Gu H. Boulting G. Smith Z. D. Ziller M. Croft G. F. Amoroso M. W. Oakley D. H. (2011). Reference maps of human ES and iPS cell variation enable high-throughput characterization of pluripotent cell lines. Cell 144**,** 439–452. 21295703 10.1016/j.cell.2010.12.032 PMC3063454

[kfu269-B8] Bone H. K. Nelson A. S. Goldring C. E. Tosh D. Welham M. J. (2011). A novel chemically directed route for the generation of definitive endoderm from human embryonic stem cells based on inhibition of GSK-3. J. Cell Sci. 124**,** 1992–2000. 21610099 10.1242/jcs.081679 PMC3104033

[kfu269-B9] Brolen G. Sivertsson L. Bjorquist P. Eriksson G. Ek M. Semb H. Johansson I. Andersson T. B. Ingelman-Sundberg M. Heins N. (2010). Hepatocyte-like cells derived from human embryonic stem cells specifically via definitive endoderm and a progenitor stage. J. Biotechnol. 145**,** 284–294. 19932139 10.1016/j.jbiotec.2009.11.007

[kfu269-B10] Chang J. Nicolas E. Marks D. Sander C. Lerro A. Buendia M. A. Xu C. Mason W. S. Moloshok T. Bort R. (2004). miR-122, a mammalian liver-specific microRNA, is processed from hcr mRNA and may downregulate the high affinity cationic amino acid transporter CAT-1. RNA Biol. 1**,** 106–113. 17179747 10.4161/rna.1.2.1066

[kfu269-B11] Chen K. Rajewsky N. (2007). The evolution of gene regulation by transcription factors and microRNAs. Nat. Rev. Genet. 8**,** 93–103. 17230196 10.1038/nrg1990

[kfu269-B12] Davies E. C. Green C. F. Mottram D. R. Rowe P. H. Pirmohamed M. (2010). Emergency re-admissions to hospital due to adverse drug reactions within 1 year of the index admission. Br. J. Clin. Pharmacol. 70**,** 749–755. 21039769 10.1111/j.1365-2125.2010.03751.x PMC2997315

[kfu269-B13] Godoy P. Hewitt N. J. Albrecht U. Andersen M. E. Ansari N. Bhattacharya S. Bode J. G. Bolleyn J. Borner C. Bottger J. (2013). Recent advances in 2D and 3D in vitro systems using primary hepatocytes, alternative hepatocyte sources and non-parenchymal liver cells and their use in investigating mechanisms of hepatotoxicity, cell signaling and ADME. Arch. Toxicol. 87**,** 1315–1530. 23974980 10.1007/s00204-013-1078-5 PMC3753504

[kfu269-B14] Greenhough S. Medine C. N. Hay D. C. (2010). Pluripotent stem cell derived hepatocyte like cells and their potential in toxicity screening. Toxicology 278**,** 250–255. 20674645 10.1016/j.tox.2010.07.012

[kfu269-B15] Harrill A. H. Roach J. Fier I. Eaddy J. S. Kurtz C. L. Antoine D. J. Spencer D. M. Kishimoto T. K. Pisetsky D. S. Park B. K. (2012). The effects of heparins on the liver: application of mechanistic serum biomarkers in a randomized study in healthy volunteers. Clin. Pharmacol. Ther. 92**,** 214–220. 22739141 10.1038/clpt.2012.40 PMC4320779

[kfu269-B16] Hay D. C. Zhao D. Fletcher J. Hewitt Z. A. McLean D. Urruticoechea-Uriguen A. Black J. R. Elcombe C. Ross J. A. Wolf R. (2008). Efficient differentiation of hepatocytes from human embryonic stem cells exhibiting markers recapitulating liver development in vivo. Stem Cells 26**,** 894–902. 18238852 10.1634/stemcells.2007-0718

[kfu269-B17] Kia R. Sison R. L. Heslop J. Kitteringham N. R. Hanley N. Mills J. S. Park B. K. Goldring C. E. (2013). Stem cell-derived hepatocytes as a predictive model for drug-induced liver injury: are we there yet? *Br**. *J. Clin. Pharmacol. 75**,** 885–896. 10.1111/j.1365-2125.2012.04360.x PMC3612706 22703588

[kfu269-B18] Kim N. Kim H. Jung I. Kim Y. Kim D. Han Y. M. (2011). Expression profiles of miRNAs in human embryonic stem cells during hepatocyte differentiation. Hepatol. Res. 41**,** 170–183. 21269386 10.1111/j.1872-034X.2010.00752.x

[kfu269-B19] Lagos-Quintana M. Rauhut R. Yalcin A. Meyer J. Lendeckel W. Tuschl T. (2002). Identification of tissue-specific microRNAs from mouse. Curr. Biol. 12**,** 735–739. 12007417 10.1016/s0960-9822(02)00809-6

[kfu269-B20] Lappalainen K. Jaaskelainen I. Syrjanen K. Urtti A. Syrjanen S. (1994). Comparison of cell proliferation and toxicity assays using two cationic liposomes. Pharm. Res. 11**,** 1127–1131. 7971713 10.1023/a:1018932714745

[kfu269-B21] Laudadio I. Manfroid I. Achouri Y. Schmidt D. Wilson M. D. Cordi S. Thorrez L. Knoops L. Jacquemin P. Schuit F. (2012). A feedback loop between the liver-enriched transcription factor network and miR-122 controls hepatocyte differentiation. Gastroenterology 142**,** 119–129. 21920465 10.1053/j.gastro.2011.09.001

[kfu269-B22] LeCluyse E. L. Alexandre E. Hamilton G. A. Viollon-Abadie C. Coon D. J. Jolley S. Richert L. (2005). Isolation and culture of primary human hepatocytes. Methods Mol. Biol. 290**,** 207–229. 15361665 10.1385/1-59259-838-2:207

[kfu269-B23] Liu D. Fan J. Mei M. Ingvarsson S. Chen H. (2009). Identification of miRNAs in a liver of a human fetus by a modified method. PLoS One 4**,** e7594. 19855840 10.1371/journal.pone.0007594 PMC2762743

[kfu269-B24] Mosmann T. (1983). Rapid colorimetric assay for cellular growth and survival: application to proliferation and cytotoxicity assays. J. Immunol. Methods 65**,** 55–63. 6606682 10.1016/0022-1759(83)90303-4

[kfu269-B25] O'Brien P. J. Irwin W. Diaz D. Howard-Cofield E. Krejsa C. M. Slaughter M. R. Gao B. Kaludercic N. Angeline A. Bernardi P. (2006). High concordance of drug-induced human hepatotoxicity with in vitro cytotoxicity measured in a novel cell-based model using high content screening. Arch. Toxicol. 80**,** 580–604. 16598496 10.1007/s00204-006-0091-3

[kfu269-B26] Olsen A. K. Whalen M. D. (2009). Public perceptions of the pharmaceutical industry and drug safety: implications for the pharmacovigilance professional and the culture of safety. Drug Saf. 32**,** 805–810. 19722724 10.2165/11316620-000000000-00000

[kfu269-B27] Rashid S. T. Corbineau S. Hannan N. Marciniak S. J. Miranda E. Alexander G. Huang-Doran I. Griffin J. Ahrlund-Richter L. Skepper J. (2010). Modeling inherited metabolic disorders of the liver using human induced pluripotent stem cells. J. Clin. Invest. 120**,** 3127–3136. 20739751 10.1172/JCI43122 PMC2929734

[kfu269-B28] Rausch O. (2006). High content cellular screening. Curr. Opin. Chem. Biol. 10**,** 316–320. 16793326 10.1016/j.cbpa.2006.06.004

[kfu269-B29] Rodriguez-Antona C. Donato M. T. Boobis A. Edwards R. J. Watts P. S. Castell J. V. Gomez-Lechon M. J. (2002). Cytochrome P450 expression in human hepatocytes and hepatoma cell lines: molecular mechanisms that determine lower expression in cultured cells. Xenobiotica 32**,** 505–520. 12160483 10.1080/00498250210128675

[kfu269-B30] Rowe C. Goldring C. E. Kitteringham N. R. Jenkins R. E. Lane B. S. Sanderson C. Elliott V. Platt V. Metcalfe P. Park B. K. (2010). Network analysis of primary hepatocyte dedifferentiation using a shotgun proteomics approach. J. Proteome Res. 9**,** 2658–2668. 20373825 10.1021/pr1001687

[kfu269-B31] Salminen W. F. Yang X. Shi Q. Mendrick D. L. (2011). Using microRNA as biomarkers of drug-induced liver injury. *J. Mol. Biomark. Diagn*. 2**,** 119.

[kfu269-B32] Sempere L. F. Freemantle S. Pitha-Rowe I. Moss E. Dmitrovsky E. Ambros V. (2004). Expression profiling of mammalian microRNAs uncovers a subset of brain-expressed microRNAs with possible roles in murine and human neuronal differentiation. Genome Biol. 5**,** R13. 15003116 10.1186/gb-2004-5-3-r13 PMC395763

[kfu269-B33] Shiraki N. Umeda K. Sakashita N. Takeya M. Kume K. Kume S. (2008). Differentiation of mouse and human embryonic stem cells into hepatic lineages. Genes Cells 13**,** 731–746. 18513331 10.1111/j.1365-2443.2008.01201.x

[kfu269-B34] Sjogren A. K. Liljevald M. Glinghammar B. Sagemark J. Li X. Q. Jonebring A. Cotgreave I. Brolén G. Andersson T. B. (2014). Critical differences in toxicity mechanisms in induced pluripotent stem cell-derived hepatocytes, hepatic cell lines and primary hepatocytes. Arch. Toxicol. 88**,** 1427–1437. 24912781 10.1007/s00204-014-1265-z

[kfu269-B35] Slater K. (2001). Cytotoxicity tests for high-throughput drug discovery. Curr. Opin. Biotechnol. 12**,** 70–74. 11167076 10.1016/s0958-1669(00)00177-4

[kfu269-B36] Soderdahl T. Kuppers-Munther B. Heins N. Edsbagge J. Bjorquist P. Cotgreave I. Jernstrom B. (2007). Glutathione transferases in hepatocyte-like cells derived from human embryonic stem cells. Toxicol. in vitro 21**,** 929–937. 17346923 10.1016/j.tiv.2007.01.021

[kfu269-B37] Starkey Lewis P. J. Dear J. Platt V. Simpson K. J. Craig D. G. Antoine D. J. French N. S. Dhaun N. Webb D. J. Costello E. M. (2011). Circulating microRNAs as potential markers of human drug-induced liver injury. Hepatology 54**,** 1767–1776. 22045675 10.1002/hep.24538

[kfu269-B38] Takahashi K. Tanabe K. Ohnuki M. Narita M. Ichisaka T. Tomoda K. Yamanaka S. (2007). Induction of pluripotent stem cells from adult human fibroblasts by defined factors. Cell 131**,** 861–872. 18035408 10.1016/j.cell.2007.11.019

[kfu269-B39] Takebe T. Sekine K. Enomura M. Koike H. Kimura M. Ogaeri T. Zhang R. R. Ueno Y. Zheng Y. W. Koike N.*.* (2013). Vascularized and functional human liver from an iPSC-derived organ bud transplant. Nature 499**,** 481–484. 23823721 10.1038/nature12271

[kfu269-B40] Ukairo O. McVay M. Krzyzewski S. Aoyama S. Rose K. Andersen M. E. Khetani S. R. Lecluyse E. L. (2013). Bioactivation and toxicity of acetaminophen in a rat hepatocyte micropatterned coculture system. J. Biochem. Mol. Toxicol. 27**,** 471–478. 23918466 10.1002/jbt.21512

[kfu269-B41] Ulvestad M. Nordell P. Asplund A. Rehnström M. Jacobsson S. Holmgren G. Davidson L. Brolén G. Edsbagge J. Björquist P. (2013). Drug metabolizing enzyme and transporter protein profiles of hepatocytes derived from human embryonic and induced pluripotent stem cells. *Biochem. Pharmacol*. 86**,** 691–702. 23856292 10.1016/j.bcp.2013.06.029

[kfu269-B42] Wang K. Zhang S. Marzolf B. Troisch P. Brightman A. Hu Z. Hood L. E. Galas D. J. (2009). Circulating microRNAs, potential biomarkers for drug-induced liver injury. Proc. Natl. Acad. Sci. U.S.A. 106**,** 4402–4407. 19246379 10.1073/pnas.0813371106 PMC2657429

[kfu269-B43] Yildirimman R. Brolen G. Vilardell M. Eriksson G. Synnergren J. Gmuender H. Kamburov A. Ingelman-Sundberg M. Castell J. Lahoz A. (2011). Human embryonic stem cell derived hepatocyte-like cells as a tool for in vitro hazard assessment of chemical carcinogenicity. Toxicol. Sci. 124**,** 278–290. 21873647 10.1093/toxsci/kfr225 PMC3216410

