# Supplementary material for: MicroRNA-122: A Novel Hepatocyte-Enriched in vitro Marker of Drug-Induced Cellular Toxicity
Source: Toxicol Sci. 2014 Dec 18;144(1):173–85. doi: 10.1093/toxsci/kfu269 (PMC4349141; doi:10.1093/toxsci/kfu269)
Supplement: Supplementary Data [file supp_144_1_173__index.html]

MicroRNA-122: a novel hepatocyte-enriched in vitro marker of drug-induced cellular toxicity — MicroRNA-122: A Novel Hepatocyte-Enriched in vitro Marker of Drug-Induced Cellular Toxicity — MicroRNA-122: A Novel Hepatocyte-Enriched in vitro Marker of Drug-Induced Cellular Toxicity — Supplementary Data 

# MicroRNA-122: A Novel Hepatocyte-Enriched *in vitro* Marker of Drug-Induced Cellular Toxicity

## Supplementary Data

files

**Files in this Data Supplement:**

- Supplementary Data - docx file
- Supplementary Data - tif file
- Supplementary Data - tif file
- Supplementary Data - tif file
