# Supplementary material for: MicroRNA-122: A Novel Hepatocyte-Enriched in vitro Marker of Drug-Induced Cellular Toxicity
Source: Toxicol Sci. 2014 Dec 18;144(1):173–85. doi: 10.1093/toxsci/kfu269 (PMC4349141; doi:10.1093/toxsci/kfu269)
Supplement: Supplementary Data [file supp_kfu269_New_Microsoft_Office_Word_Document.docx]

**Supplementary Figure 1. Analysis of the change in the number of copies of miR-122 in the lysate and media components of human primary hepatocyte cultures treated with acetaminophen and diclofenac.**

Data is presented as the mean +/- SEM of the number of copies of miR-122 in the lysate and/or media components of the dose-response experiments of human primary hepatocytes treated with (A) acetaminophen and (B) diclofenac, from 3 independent experiments using different donors of human hepatocytes. The number of copies of miR-122 estimated in the media and lysates were normalised to the number of hepatocytes in the culture to allow for a direct comparison of absolute quantities of miR-122 in the media with other cell types used for similar cytotoxicity assays. The cytotoxicity assays were performed on hepatocytes plated in 24-well plates at a density of 5 x 10^5^ hepatocytes per well. The same raw values from these graphs were used to calculate the percentage of total miR-122 in the media shown in Figure 2.

**Supplementary Figure 2. Analysis of the change in the number of copies of miR-122 over time in the lysate and media components of human primary hepatocyte cultures treated with non-toxic and toxic concentrations of diclofenac.**

Data is presented as the mean number of copies of miR-122 in the lysate and/or media components of the time-course experiments of (A) Untreated human primary hepatocytes, and hepatocytes treated with (B) non-toxic concentration of 0.1 mM diclofenac and (C) toxic concentration of 1 mM diclofenac. Error bars indicate the SEM from 3 independent experiments using different donors of human primary hepatocytes. The same raw values from these graphs were used to calculate the percentage of total miR-122 in the media shown in Figure 3.

**Supplementary Figure 3. Correlation between absolute quantification of miR-122 copies in the media and the relative percentage levels of miR-122 and LDH in the media of human primary hepatocytes treated with acetaminophen and diclofenac**

Experimental values from the dose-response experiments of human primary hepatocytes treated with acetaminophen and diclofenac for 24 hours (Figure 2) were used to examine the correlation of mean number of copies of miR-122 with (A) mean percentage of total miR-122 and (B) mean percentage of total LDH, in the media. r denotes Pearson’s correlation coefficient, CI denotes the confidence interval of r.
